# Amphioxus mouth after dorso-ventral inversion

**DOI:** 10.1186/s40851-016-0038-3

**Published:** 2016-02-06

**Authors:** Takao Kaji, James D. Reimer, Arseniy R. Morov, Shigeru Kuratani, Kinya Yasui

**Affiliations:** Department of Biological Science, Graduate School of Science, Hiroshima University, 1-3-1 Kagamiyama, Higashi-hiroshima, Hiroshima 739-8526 Japan; Present address: Department of Diabetes Technology, Graduate School of Biomedical Engineering, Tohoku University, 6-6-12 Aramaki Aza Aoba, Aoba-ku, Sendai, Miyagi 980-8579 Japan; Department of Biology, Chemistry and Marine Sciences, Faculty of Science, University of the Ryukyus, 1 Senbaru, Nishihara, Okinawa 903-0213 Japan; Department of Zoology and General Biology, Institute of Fundamental Medicine and Biology, Kazan (Volga Region) Federal University, 18 Kremlyovskaya St., Kazan, 420008 Republic of Tatarstan Russian Federation; Evolutionary Morphology Laboratory, RIKEN, 2-2-3 Minatojima-minami, Chuo-ku, Kobe, Hyogo 650-0047 Japan

**Keywords:** Lancelet, Homology of mouth, Coelom, Hydropore, Nodal-signaling, Gill (branchial) slits

## Abstract

**Introduction:**

Deuterostomes (animals with ‘secondary mouths’) are generally accepted to develop the mouth independently of the blastopore. However, it remains largely unknown whether mouths are homologous among all deuterostome groups. Unlike other bilaterians, in amphioxus the mouth initially opens on the left lateral side. This peculiar morphology has not been fully explained in the evolutionary developmental context. We studied the developmental process of the amphioxus mouth to understand whether amphioxus acquired a new mouth, and if so, how it is related to or differs from mouths in other deuterostomes.

**Results:**

The left first somite in amphioxus produces a coelomic vesicle between the epidermis and pharynx that plays a crucial role in the mouth opening. The vesicle develops in association with the amphioxus-specific Hatschek nephridium, and first opens into the pharynx and then into the exterior as a mouth. This asymmetrical development of the anterior-most somites depends on the Nodal-Pitx signaling unit, and the perturbation of laterality-determining Nodal signaling led to the disappearance of the vesicle, producing a symmetric pair of anterior-most somites that resulted in larvae lacking orobranchial structures. The vesicle expressed *bmp2/4*, as seen in ambulacrarian coelomic pore-canals, and the mouth did not open when Bmp2/4 signaling was blocked.

**Conclusions:**

We conclude that the amphioxus mouth, which uniquely involves a mesodermal coelomic vesicle, shares its evolutionary origins with the ambulacrarian coelomic pore-canal. Our observations suggest that there are at least three types of mouths in deuterostomes, and that the new acquisition of chordate mouths was likely related to the dorso-ventral inversion that occurred in the last common ancestor of chordates.

## Introduction

The mouth opening is evolutionarily related to the blastopore, the first opening to connect the gut and exterior during development. In cnidarians, gastrulation occurs at the animal pole and the blastopore directly gives rise to the mouth/anus [1]. In contrast, in most bilaterians, the blastopore forms at the vegetal pole, and primarily determines the anteroposterior body axis [2]. As the blastopore is at the posterior in their developmental system, bilaterians exhibit various patterns of mouth formation in the anterior body.

Some protostomes utilize the blastopore as the mouth after shifting the blastopore towards the anterior during development [3, 4], which is referred to as protostomy. In contrast, deuterostome ambulacrarians (echinoderms + hemichordates) and chordates open their mouths independently of the blastopore, a process called deuterostomy. The new setting of the oral site in ambulacrarian larvae is suggested to be caused by the separation of mouth-forming gene regulatory networks (GRN) from the ancestral blastoporal GRN that specifies mouth formation, axis determination, and germ layer specification [5]. This uncoupling with a gene set comparable to ambulacrarians is also found in protostome animals [5, 6] and thus seems to represent an ancestral state.

**Fig. 1:**
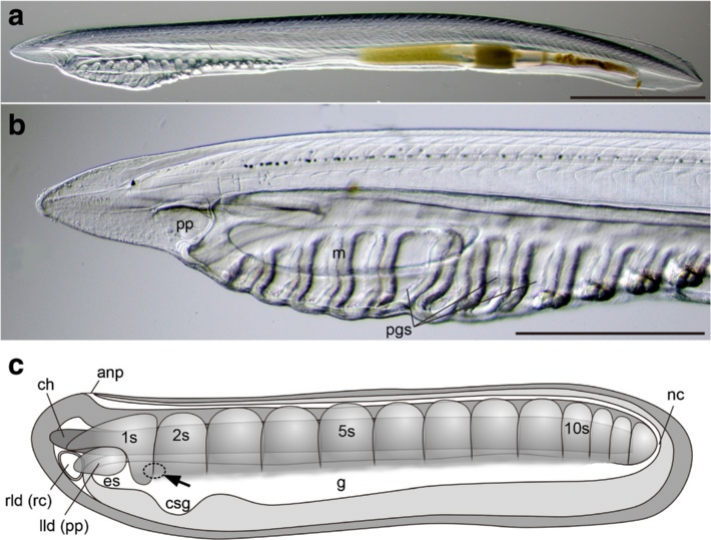
Larval asymmetry of amphioxus. **a** Left lateral view of premetamorphic larva showing streamlined shape. **b** Magnification of oral region showing mouth and preoral pit on left and gill openings on right side. **c** Schematic illustration of early knife-shaped larva showing organ formation in anterior region and previously suggested location of mouth opening (*arrow*). anp; anterior neuropore; ch, notochord; csg, primordial club-shaped gland; es, primordial endostyle; g, gut; lld, left lateral diverticulum (= pp, preoral pit); m, mouth; nc, neurenteric canal; pgs, primary gill slit; rld, right lateral diverticulum (= rc, rostral coelom); 1-10s, 1st-10th somite. Scale bars, 1.0 mm for (**a**), 0.5 mm for (**b**)

Vertebrates, however, do not share the ancestral mouth-forming GRN with other animals [7], and also have an interesting dorso-ventral axis characterized by Bmp expression on one side and Chordin, a repressor of Bmp signaling, expression on the other. The vertebrate mouth opens on the Bmp-expressing side, whereas non-chordate bilaterians have mouths on the Chordin-expressing side [8]. Among chordates, amphioxus exhibits a further unique pattern, in which the mouth initially opens on the left, despite a streamlined body shape (Fig. 1a, b). This asymmetrical mouth opening appears at early larval stages along with other pharyngeal structures, such as the lateral diverticula, mucus-secreting glands, and gill openings (Fig. 1c). The unusual location of the amphioxus larval mouth is a longstanding enigma, and various hypotheses have been put forth in an effort to homologize the amphioxus mouth to structures in other animals. (1) The amphioxus mouth is derived from the first left gill pore, thereby being homologous to the vertebrate spiracle [9–11], (2) it is homologous to the mid-ventrally located mouth of most bilaterians, which in amphioxus moved to the left side due to adaptation from a proposed past epibenthic life mode [12], or (3) it is homologous to the mid-dorsally located larval ascidian mouth that in amphioxus has moved to the left due to anterior elongation of the notochord [13, 14]. The last two hypotheses are based on the idea that all animal mouths are homologous. It has also been suggested, alternatively, that the amphioxus mouth is a novel trait uniquely acquired in the cephalochordate lineage [15]. All of these ideas are attractive but remain unverified.

In this study, we identified a coelomic vesicle that developed from the posterior wall of the left anterior-most somite. This vesicle accompanies the Hatschek nephridium and contributes to the formation of the larval mouth. The oral development of amphioxus was controlled under the evolutionarily conserved Nodal-Pitx signaling unit [16–18] on the left side and has no relation to the mouth-forming GRN found in ambulacrarians and protostomes. The developmental process of the amphioxus mouth strongly suggests its similarity to that of ambulacrarian coelomic pore-canals and their common evolutionary origin. Thus the amphioxus mouth is distinct from olfactorean (urochordates and vertebrates) [19] mouths that develop from a specific region called the anterior pan-placode at the anterior extremity of the body [7] or its equivalent [20]. We suggest that deuterostomes display at least three types of different mouths, of which at least two types have been acquired by chordates, all developing independently of the blastopore.

## Materials and methods

### Experimental animals

Amphioxus specimens studied in the present research were from a Japanese population of *Branchiostoma japonicum* (formerly *B. belcheri*) [21, 22]. All embryos and larvae were collected during breeding season from first laboratory generations bred by parental animals collected from a wild habitat in the Ariake Sea, Japan, that were then maintained in a laboratory culture system [22]. The amphioxus colony is maintained and all embryos and larvae subjected to the present study were manipulated according to guidelines established by Hiroshima University for the care and use of experimental animals.

### Preparation of RNA probes for whole-mount in situ hybridization

In total seven DNA fragments encoding the coding region of seven genes were amplified by PCR with primer sets listed in Table 1. Template cDNA for PCR was constructed from a frozen sample of spawn at the neurula stage with 7–9 somites by using ISOGEN (Nippon Gene, Japan) for isolating total RNA, and SMARTer RACE cDNA Amplification Kit (Clonetech, California) for constructing first strand cDNA. Amplicons were cloned into the pGEM-T Easy vector (Promega, Wisconsin) and sequenced to confirm identity. Digoxigenin- or fluorescein-labeled antisense riboprobes were synthesized by SP6 or T7 RNA polymerase (Roche Applied Science, Germany).

**Table 1:** List of forward and reverse primer sets for PCR amplification

Name of gene	Forward primer	Reverse primer	Accession no.	Reference
*dkk1/2/4*	CCGTAACTTGTCCACACCGTAGA	GTGCTTTGATGCGTCTTGTTGGG	HM590023	Zhang and Mao (2010) [35]
*frzb1* ^a)^	GTGGTTCCTCCCGTTGTTG	GCTTCAGCCGTCTTTCCCA	XM002612836	Putnam et al. (2008) [52]
*lim1/5* ^b)^	AGGGACTCCAAACTGTACTG	TTACCACACCGACTCTTCC	DQ399521	Langeland et al. (2006) [30]
*mef2* ^c)^	GCTTATGAGCTGAGTGTGCT	GGTTCACTCTTGATCTGCAT	EF407505	Zhang et al. (2007) [53]
*pax2/5/8*	TGTGACAACGACACAGTTCC	AACAGCAACTGGATAGTGGC	AB193513	Hiruta et al. (2005) [54]
*pax3/7* ^d)^	GGTGGAGAAGAAGATAGAGG	ACGGATGTTCAGTACGCTTG	XM002610760	Holland et al. (1999) [55]
*pou4* ^e)^	ACAACACATCAGCATGCACC	GGTTTTGTTTTGACCGTTATT	DQ314242	Candiani et al. (2006) [28]

Sequences of *Branchiostoma japonicum* gene fragments were registered under accession numbers ^a)^AB979875, ^b)^AB980198, ^c)^AB980199, ^d)^AB980200, and ^e)^AB980202

### Whole-mount in situ hybridization (WISH)

Embryos and larvae for WISH were fixed at 4 °C overnight with freshly prepared 4 % paraformaldehyde in 0.1 M 3-(N-morpholino) propanesulfonic acid (MOPS) buffer, pH 7.5, containing 0.5 M NaCl, then washed with 50 % ethanol, and stored in 75 % ethanol at -20 °C until use. WISH was performed with a setting temperature of 60 °C for prehybridization and hybridization according to previously described protocol [23]. Expression patterns were observed after changing solution from phosphate buffered saline (PBS, pH 7.4) to 80 % glycerol in PBS. To obtain rendering images of confocal laser scanning microscopy (LSM), double fluorescence WISH was performed with anti-sense riboprobes for *mef2* and *pou4* or *pax3/7.* Anti-DIG-POD (Roche Applied Science, Germany) and anti-fluorescein-POD (PerkinElmer, Massachusetts) antibodies were used to detect the anti-sense riboprobes and then the TSA Plus Cyanine 3 & Fluorescein system (PerkinElmer, Massachusetts) was utilized to amplify the fluorescent signal [24]. For diffusion of labeled fluorescein into the whole body, labeled specimens were stored in 80 % glycerol in PBS for more than one month in dark at 4 °C. Subsequently, rendering images were obtained by using an LSM (ECLIPS C1, Nikon, Japan).

### Semi-thin plastic sections and transmission electron microscopy (TEM)

Larvae for TEM were fixed with 1.25 % glutaraldehyde and 1 % paraformaldehyde in Millipore filtered seawater (MPFSW) at 4 °C overnight. Fixed specimens were washed twice with MPFSW and stored in MPFSW with a few drops of the same fixative as for WISH at 4 °C. After washing with MPFSW, specimens were subjected to a conductive staining with 1 % tannic acid in MPFSW for 30 min at 4 °C, washed with MPFSW, and then postfixed with 1 % osmium tetroxide for 1 hour at 4 °C. Postfixed specimens were dehydrated conventionally, then passed through pure propylene oxide, and finally embedded into Epon 812 following a previous study [25]. Knife-shaped larvae fixed at various times (24-30 hours post fertilization (hpf) at 25 °C) were transversely serially cut at 1-μm thickness and stained with toluidine blue to optically observe the developmental changes of the anterior part of the body. Based on observation of the semi-thin sections, appropriate specimens in resin were trimmed into blocks for TEM observation with a JEM1400 (JEOL, Japan) at Hiroshima University.

### Fluorescent immunostaining

Specimens for immunostaining were obtained from those fixed for WISH. Antibodies used for detecting nerves, ventral muscles, and basal laminae were anti-acetylated tubulin antibody (T6793, Sigma-Aldrich, Missouri), anti-αSM1 antibody (NCL-SMA, Leica, UK), and anti-laminin antibody (L9393, Sigma-Aldrich, Missouri), respectively. The antibodies were diluted to 1/200 with Can Get Signal (Toyobo, Japan). As secondary antibodies, anti-mouse or -rabbit IgG antibodies labeled with Alexa Fluor 488 or 594 (Life Technologies, California) were used at 1/500 dilution. Specimens immunoreacted with the anti-laminin antibody were also stained DNA with Hoechst (Life Technologies, California) at 5 μg/ml while washing the secondary antibody. Fluorescent images were obtained as optical sections or rendering images that covered more than half an amphioxus body width by using an LSM (ECLIPS C1, Nikon, Japan).

### Pharmacological treatments

To block Nodal signaling, embryos were treated with SB505124 (S4696, Sigma-Aldrich, Missouri) [26] at 5 μM in MPFSW from the prehatching early neurula stage to the hatching neurula stage (2 hours from 10 hpf at 25 °C). The treatment was stopped by washing twice with MPFSW. Dorsomorphin (045-31221, Wako, Japan) was used to perturb BMP signaling before the mouth opened [27]. The treatments at 5-, 10-, and 25-μM concentrations were started at the knife-shaped larval stage (24 hpf at 25 °C) and stopped at 30 hpf. Treated specimens and those reared in MPFSW with the same volume of dimethyl sulfoxide added were fixed under the same protocol as for WISH specimens at 16, 24, 36, and 66 hpf for Nodal-blocked specimens and 72 hpf for BMP-blocked specimens.

### Image data processing

Digital images in JPEG or TIFF format were obtained by an optical microscope (TE2000, Nikon, Japan), LSM (ECLIPS C1, Nikon, Japan), or TEM (JEM1400, JEOL, Japan). They were visually optimized and edited with Adobe Photoshop and Illustrator CS6 (Adobe, California).

## Results and discussion

**Fig. 2:**
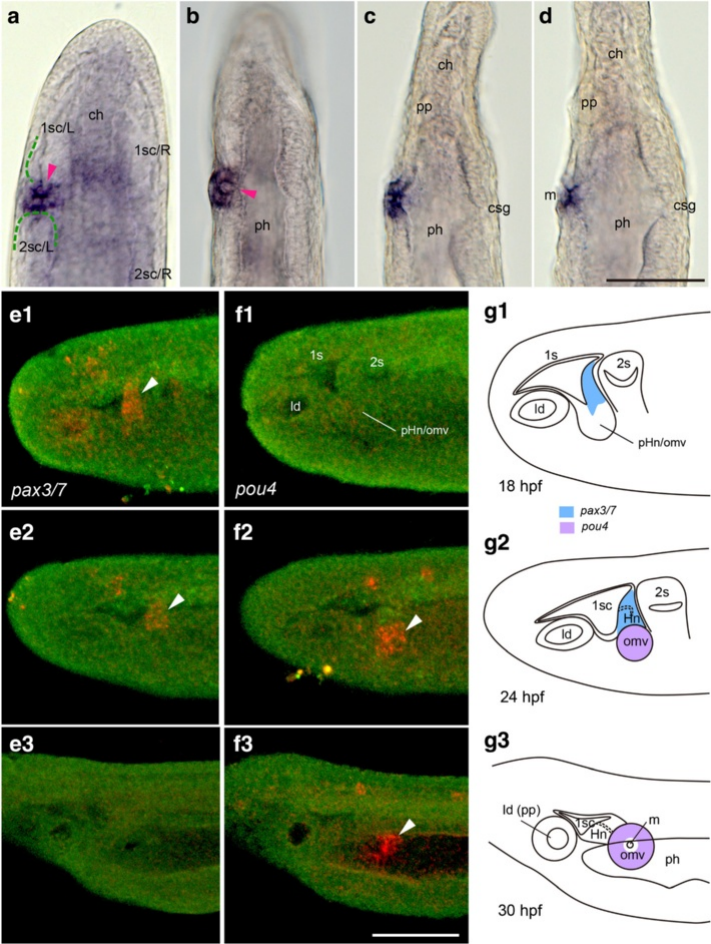
Developmental pattern of oral mesovesicle. **a**–**d** Mouth formation visualized by *pou4* expression. Oral mesovesicle initially connects with first somitocoel (arrowhead in **a**) and then into pharynx (arrowhead in **b**). Perforation of mouth occurs through epidermis and remnant of oral mesovesicle (**c**, **d**). **e**–**g** Rendering images of laser confocal microscopic analyses showing expression patterns of *pax3/7* and *pou4*. **e1**–**3** Anterior to the left. Expression of *pax3/7* (orange) in thickened posterior wall of the first somite (arrowheads) from 18 to 30 hpf at 25 °C. **f1**–**3** Expression of *pou4* (orange) in bulged wall of the first somite, which transforms into oral mesovesicle (arrowheads) from 18 to 30 hpf at 25 °C. **g1**–**3** Outlines of rendering images highlighting expression of two genes. ch, notochord; csg, club-shaped gland; ld, lateral diverticulum; m, mouth; ph, pharynx; pp, preoral pit; 1, 2 s, 1st and 2nd somite; 1, 2sc/L or R, left or right first and second somitocoel; pHn/omv, primordium of Hatschek nephridium/oral mesovesicle complex. Scale bars 50 μm

**Fig. 3:**
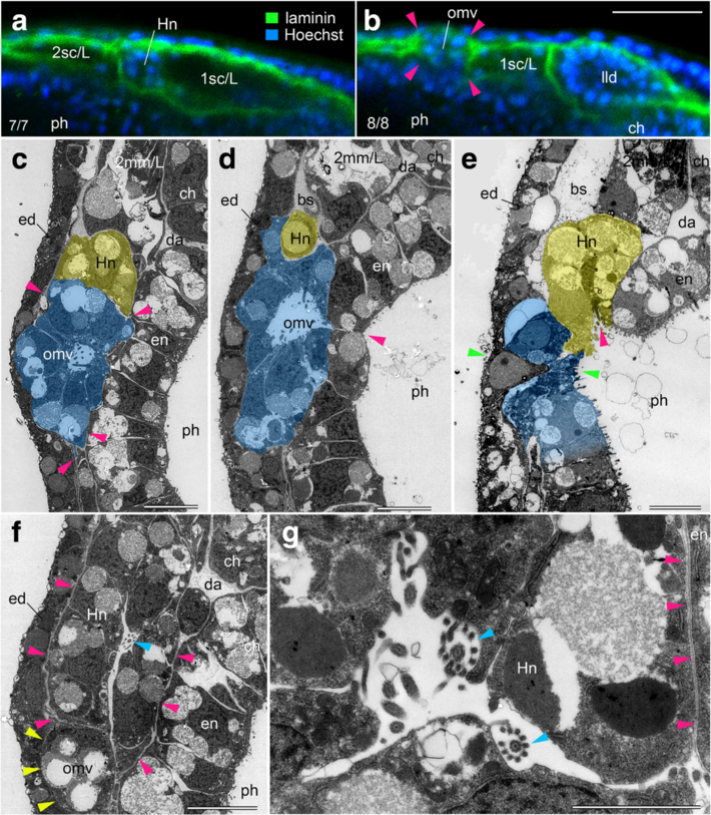
Oral mesovesicle/Hatschek nephridium complex and basal laminae. **a**, **b** Fluorescent horizontal sections showing nuclei and immunolabeling for basal laminae. Note dissolution of basal laminae on oral mesovesicle (arrowheads in **b**). **c**–**e** Electron micrographs showing transverse sections of developing oral mesovesicle. While primordium of Hatschek nephridium develops basal laminae, oral mesovesicle does not (arrowheads in **c**). Oral mesovesicle first opens into pharynx without epithelical intercalation (arrowhead in **d**). Outlet of Hatschek nephridial canal into pharynx (magenta arrowhead in **e**) and site of oral perforation (green arrowheads in **e**). **f** Electron micrographs showing transverse sections of Hatschek nephridium with flagellum and whorl of microvilli from a cyrtopodocyte (cyan arrowhead) and basal laminae (magenta arrowheads). There is no basal lamia around oral mesovesicle (yellow arrowheads). **g** Magnification of canal of Hatschek nephridium showing two flagella with a whorl of microvilli (cyan arrowheads) and basal lamina between Hatschek nephridium and pharyngeal endoderm (magenta arrowheads). bs, blood sinus; ch, notochord; da, dorsal aorta; ed, epidermis; en, endoderm; Hn, Hatschek nephridium; lld, left lateral diverticulum; omv, oral mesovesicle; ph, pharynx; 2 mm/L, left 2nd myomere;1-2sc/L, left 1st and 2nd somitocoel. Scale bars 20 μm for (**a**), (**b**), 5 μm for (**c**–**f**), and 2 μm for (**g**)

To identify the positional specification for mouth opening, we initially focused on the expression of the transcription factor gene *pou4*, which is reported to be expressed in the oral region including within a cell mass under the epidermis at the early larval stage, as well as at the margin of the mouth during perforation [28]. We observed a vesicle between the left first and second somite in optical horizontal sections of larvae that visualized *pou4* expression, confirming that the *pou4-*expressing subepidermal cell mass is a vesicle (Fig. 2a, b). By tracing *pou4* expression during early larval development, we confirmed a connection between the vesicle and the left first somite at the earliest larval stage (Fig. 2a, b). The vesicle was subsequently isolated from the somitic cavity and opened into the pharynx. The vesicle was then incorporated into the pharyngeal endoderm while maintaining tight contact with the epidermis (Figs. 2c, d and 3d, e). The perforation of the mouth occurred between the epidermis and the vesicle remnant that still expressed *pou4* (Fig. 2d).

To further understand the developmental process of the vesicle formation, we performed fluorescent WISH with *pax3/7,* an upstream muscle specification gene [29], and *pou4* probes for a developmental series of early larvae. Fluorescent rendering images of LSM clarified that the vesicle developed from the posterior ventral corner of the left first somite (Fig. 2e–g). The posterior wall first became thickened and bulged ventrally at the late neurula stage (18–20 hpf at 25 °C). The thickened wall expressed *pax3/7*, and subsequently the ventral bulge was extruded from the wall, incorporating the somitocoelic lumen as a vesicle, and began to express *pou4* (Fig. 2e–g). In the *pax3/7*-expressing region, a canal newly formed to connect the somitocoel and cavity of the vesicle. These observations confirmed that this vesicle (hereafter referred to as oral mesovesicle, OMV) is mesodermal as previously suggested [30, 31], and that the OMV is the definitive source of the larval mouth in amphioxus.

Within the above-noted canal, TEM observations identified cell(s) bearing a flagellum with a whorl of microvilli, a character of amphioxus excretory cells called cyrtopodocytes [32] (Fig. 3f, g). The canal initially opened into the pharynx through the cavity of the OMV. Our observations highlight the close developmental relationship between the Hatschek nephridium (Hn) and the mouth in amphioxus, although classical studies inferred that the mouth opened independently from the bulge of the left first somite even as they described the bulge [9, 33].

The primordial oral site in vertebrate embryos commonly appears as a median pan-placode at the anterior extremity of the body, and the pan-placode contains the olfactory, pituitary, and stomodaeum subdomains from the dorsal to ventral direction [7]. The stomodeal ectoderm is directly in contact with the underlying endoderm without any intervening mesodermal cells, and dissolves the basal lamina between itself and the pharyngeal endoderm to form an oropharyngeal membrane that opens to the exterior as a mouth [7]. Since the dissolution of basal laminae is essential for fusion and intercalation between the two epithelia, we examined this by TEM observations and immunolabeling with an anti-laminin antibody.

When the OMV became an isolated vesicle, it was tightly in contact with both the epidermis and endoderm without any basal laminae as in the vertebrate stomodaeum, as confirmed by the TEM observations and immunostaining against laminin (Fig. 3a–c, f, g). The cell mass of the Hatschek nephridium mounted the OMV and developed basal lamina on the surface except in the area in contact with the OMV (Fig. 3c, f). The perforation between the OMV and pharynx occurred without intercalation of the two epithelia, but the OMV appeared to be incorporated into the pharyngeal endoderm (Fig. 3d, e). At the final stage, intercalation occurred between the thinned epidermis and the remnant of the OMV that was regionally restricted to the future perforation site. The opening of the Hatschek nephridium was located dorsal to the mouth opening site (Fig. 3e).

**Fig. 4:**
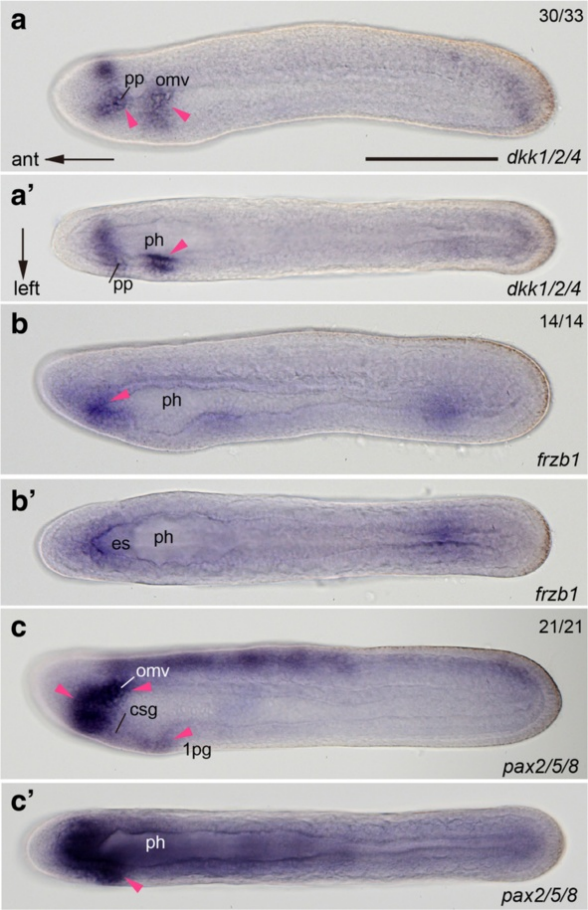
Expression patterns of antagonistic genes to Wnt and pax2/5/8 related to dissolution of basal laminae. **a**–**c** Left lateral views of knife-shaped larvae. **a**’–**c**’ Dorsal views. Most genes are expressed in the region where internal lumens open into the exterior. Arrowheads denote gene expression regions. csg, club-shaped gland; es, endostyle; omv, oral mesovesicle; 1 pg, future first gill; ph, pharynx; pp, preoral pit. Scale bar, 100 μm for all

It has been reported that basal laminae in the oral region are positively controlled by Wnt signaling in *Xenopus*, whereas Wnt antagonists such as Dickkopf (Dkk) and secreted Frizzled-related proteins function in its dissolution [34]. To determine whether the formation of basal laminae around the OMV is regulated by similar molecular mechanisms to those of vertebrates, we analyzed the expression of the corresponding genes *dkk1/2/4* [35] and *frzb1* in *Branchiostoma japonicum.* Wnt antagonist-encoding genes were expressed in the left lateral diverticulum and OMV with some extension into surrounding regions (Fig. 4). Although these Wnt antagonists are secreted proteins and the expression domains of the genes encoding these proteins did not show any clear boundaries, the basal laminae in the Hn/OMV complex clearly demarcated between these two components in TEM images (Fig. 3c, f). The Pax2 protein has also been suggested to have a role in the dissolution of basal laminae [36], and amphioxus gene *pax2/5/8* was expressed in the lateral diverticula, OMV, and gills before perforation (Fig. 4 and ref. [37]). These expression patterns suggest that these anterior-specific genes commonly function in epithelial perforations in the chordate head.

**Fig. 5:**
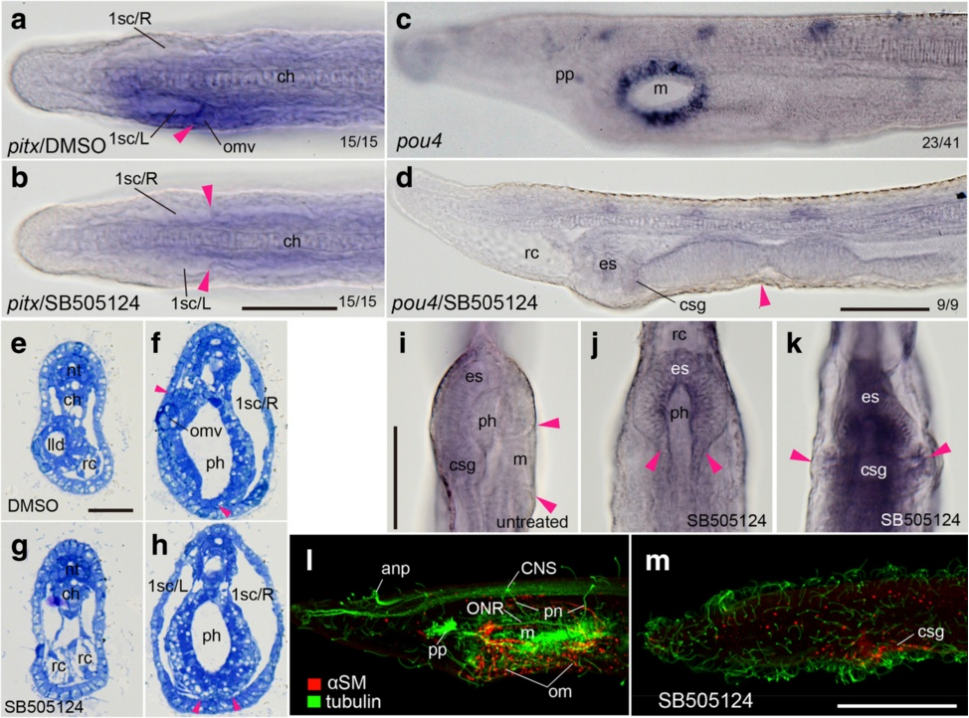
Bilaterally symmetric development of larvae treated with Nodal signaling inhibitor. **a** Dorsal view showing *pitx* expression on left side (arrowhead) of untreated 24-hpf larva. **b** Lack of left-handed *pitx* expression and symmetrical arrangement of somitocoels (arrowheads) in treated larva. **c** Mouth opening shown with *pou4* expression in control 72-hpf larva. **d** Disappearance of mouth-related expression of *pou4* and complete lack of organs originating from left side and domed pharyngeal floor (arrowhead) in treated larva. **e**, **f** Transverse sections showing future oral regions of control larvae at 24 hpf. **g**, **h** Transverse sections of treated 24-hpf larva showing bilaterally symmetric internal structures with a pair of rostral coeloms. Levels are comparable between (**e**) and (**g**) and (**f**) and (**h**), respectively. No oral mesovesicle but somitocoel on left side almost identical to that on right side in treated larvae (compare arrowheads between **f** and **h**). **i** Ventral view of normal pharynx with mouth opening (arrowheads). **j**, **k** Ventral views of treated pharynx showing bilaterally symmetric endostyle and club-shaped gland (arrowheads). **i**–**k** are shown with background of BM purple to clarify morphological details. **l**, **m** Immunolabeling for ventral muscles and acetylated tubulin. Left lateral views showing untreated larvae (72 hpf) (**l**) and SB505124-treated larvae with lack of neurons and oral musculature (66 hpf) (**m**). anp, anterior neuropore; csg, club-shaped gland; ch, notochord; CNS, central nervous system; es, endostyle; lld, left lateral diverticulum; m, mouth; nt; nerve cord; om, oral muscle; omv, oral mesovesicle; ONR, oral nerve ring; ph, pharynx; pn, peripheral nerve; pp, preoral pit; rc, rostral coelom; 1sc/L or R, first left or right somitocoel. Scale bars 50 μm for (**a**–**d**), 20 μm for (**e**–**h**), 100 μm for the others

When the pan-placode in vertebrates and the median stomodaeum in ascidians are specified, the transcription factor gene *pitx* is commonly involved [20, 38]. Amphioxus embryos also express *pitx* very weakly and transiently in the epidermis anterior to the anterior margin of the future neural plate [23]. However, amphioxus embryos rapdily express this gene asymmetrically in the future left first somite, after which the expression expands into surrounding tissues including the overlying epidermis [23]. The *pitx* expression domain includes the left first somite that develops the OMV and the oral ectoderm. The expression domain of the *nodal* gene partially overlaps that of *pitx* [39], and this colocalization suggests their interaction in amphioxus embryos. Moreover, the Nodal-Pitx signaling unit is highly conserved in animals [16, 17]. We therefore examined the role of *pitx* gene by blocking Nodal signaling at the prehatching neurula stage (10 hpf at 25 °C) with SB505124 [26]. The treated animals lacked the left-handed expression of *pitx*, but weak, bilaterally symmetrical signals in the pharynx were found (Fig. 5a, b). The treated animals also displayed a bilaterally symmetrical anatomy (Fig. 5a–d). A similar bilaterally symmetrical morphology has also been reported for *B. floridae* and *B. lanceolatum* [18, 40]. The endostyle and club-shaped gland, which develop on the right side at the anterior end of the normal pharynx as mucus secreting glands, became bilaterally symmetric. In contrast, structures that normally develop on the left side, such as the preoral pit, the outlet of the club-shaped gland, and the mouth, were entirely absent (Fig. 5c, d, i–k). Histological observations of the treated larvae confirmed that the first somites on both sides were identical. The left first somite produced neither OMV nor Hn, but instead extended towards the ventral midline and produced the left rostral coelom as the right-side counterpart normally would do (Fig. 5e–h). In 72-hpf larvae that normally developed well-defined mouths, there were no traces of mouth or gill pore development, and instead the pharyngeal floor became domed when treated with the inhibitor (Fig. 5c, d). Although normal larvae develop an oral nerve plexus and muscles [41, 42], neither muscular nor neuronal development was immunohistochemically detected in the oral region of the treated larvae (Fig. 5l, m).

**Fig. 6:**
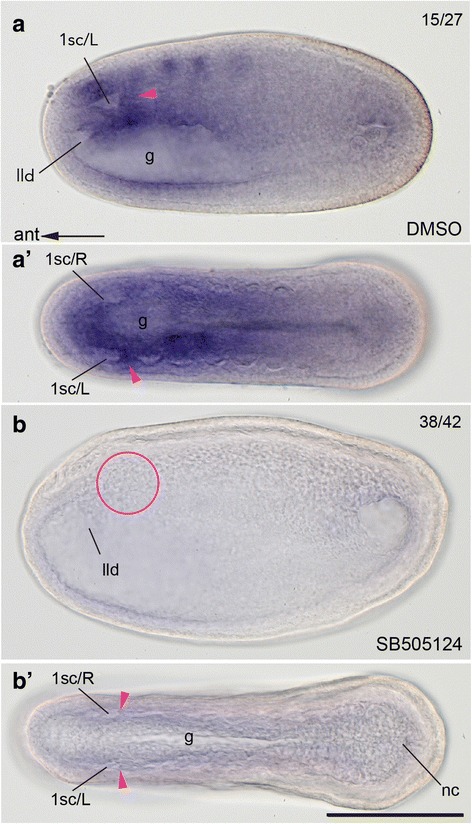
Loss of nephrogenic capability in embryos treated with Nodal signaling inhibitor. **a**, **a**’ Left lateral (**a**) and dorsal (**a**’) views of control hatched-neurula showing *lim1/5* expression in posterior wall of left first somite (arrowheads). **b**, **b**’ Loss of corresponding expression in treated neurula (circle and arrowheads) viewed left laterally (**b**) and dorsally (**b**’). g, gut; lld, left lateral diverticulum; nc, neurenteric canal; 1sc/L or R, first left or right somitocoel. Scale bar, 100 μm for all

The interruption of the Nodal signaling resulted in the complete absence of the Hn and OMV, and thus we investigated the developmental capability of the Hn/OMV complex by examining expression patterns of nephridium specific gene *lim1/5* [30]. While control neurula embryos (16 hpf at 25 °C) expressed *lim1/5* in the posterior wall of the left first somite, treated embryos showed no expression in the same region (Fig. 6). This suggests that the formation of the mouth and Hn are controlled by the left-handed expression of *pitx* or widely downstream target genes of the Nodal signaling.

**Fig. 7:**
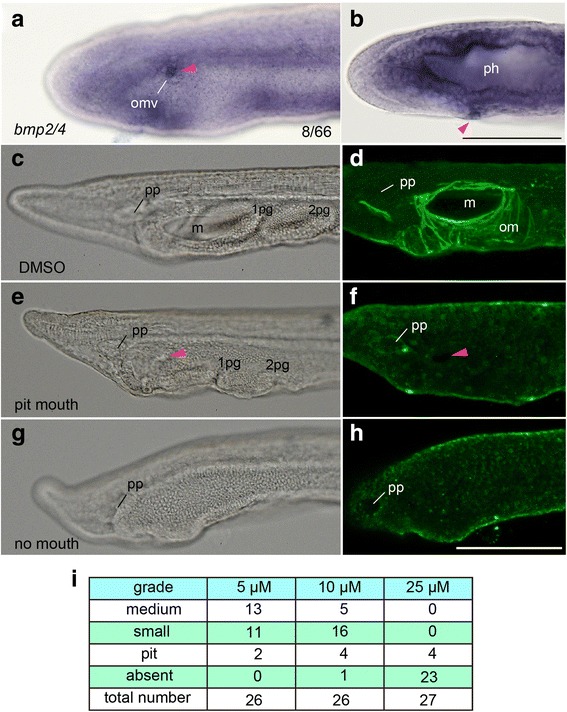
Expression of *bmp2/4* at oral mesovesicle and lack of oral structures in Dorsomophin-treated larvae. **a**, **b** Expression of *bmp2/4* at future perforation site in oral mesovesicle (arrowhead) at 24 hpf at 25 °C viewed left laterally (**a**) and dorsally (**b**). **c** Control 72-hpf larva having well-developed mouth on the left side. **d** Muscle fibers of oral musculature immunolabelled with anti-α smooth muscle actin antibody in untreated 72-hpf larva. **e**, **f** 72-hpf larva treated with Dorsomorphin reduced size of mouth as a pit (arrowhead in **e**) and absence of oral musculature (**f**). **g**, **h** Treated larva lacking mouth (**g**) and oral musculature (**h**). **i** A dose-dependent effect of Dorsomorphin on mouth opening treated from 24 to 30 hpf at 25 °C. m, mouth; om, oral musculature; 1, 2 pg, first and second primary gill; pp, preoral pit. Scale bar 100 μm for (**a**), (**b**) and the others

Ambulacrarian coeloms are reported to contribute to excretion, and thus the coelom needs to open to the exterior [43]. Ambulacrarians extend a canal or a pair of canals from a coelom to form a coelomic pore-canal (hydropore) unilaterally or bilaterally [43]. The ambulacrarian coelomic pore-canal expresses *bmp2/4* [44], and the hydropore does not form in sea urchins if Bmp2/4 signaling is blocked by the inhibitor Dorsomorphin [45]. *B. japonicum* also expressed *bmp2/4* transiently in a small region of the OMV in tight contact with the epidermis (Fig. 7a, b). We treated larvae with Dorsomorphin prior to the mouth opening and found that this inhibitor also affected the mouth formation in amphioxus in a dose-dependent manner. Like the hydropore of sea urchins, the treatment led to no mouth opening and no oral muscle development (Fig. 7c–i).

**Fig. 8:**
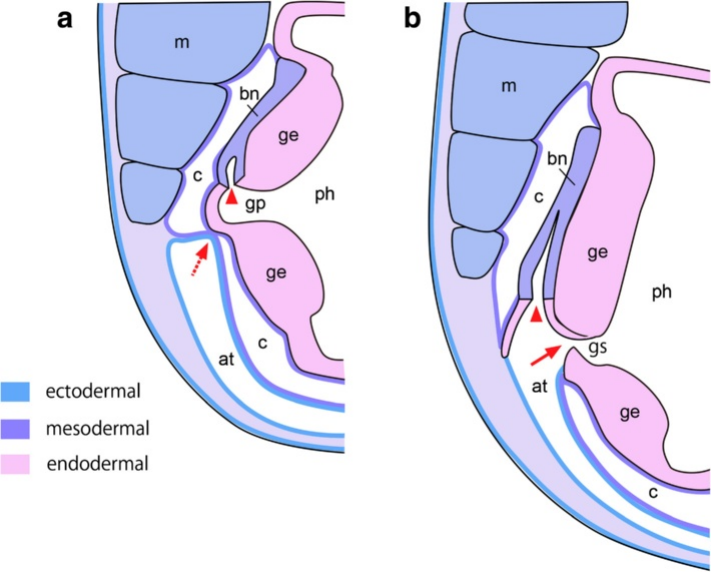
Schematic drawing of paired gill perforation in amphioxus. **a** Transverse section showing opening of branchial nephridium into pharynx (arrowhead) at which gill perforation occurs at the dorsal end of atrium (arrow). **b** Gill opens through coelomic mesothelia on both ectodermal and endodermal sides (arrow). This gill formation is comparable to mouth formation. Redrawn from ref. 50. at, atrium; bn, branchial nephridium; c, coelom; ge, gill epithelium; gp, gill pouch; gs, gill slit; m, myomere; ph, pharynx

**Fig. 9:**
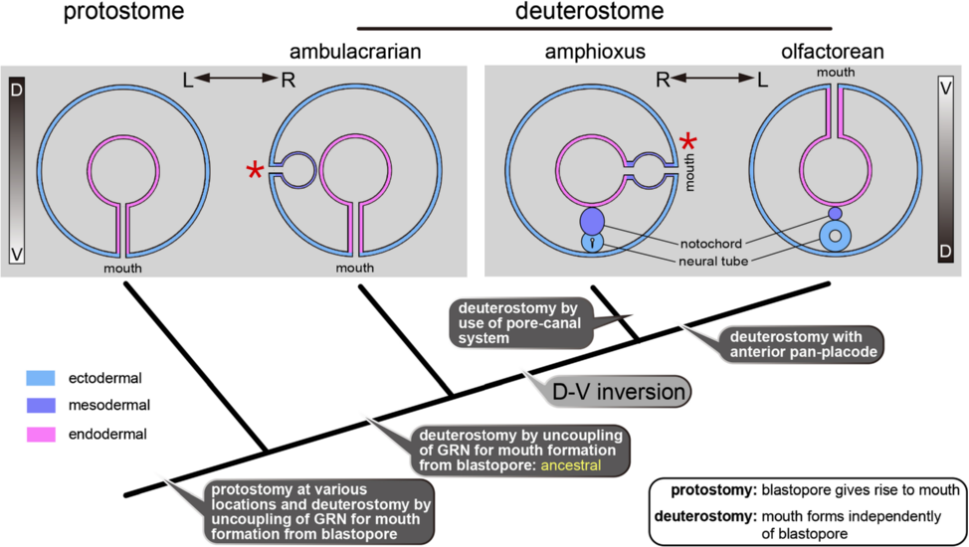
Mouth formation in deuterostome clade. Ambulacrarians retain ancestral deuterostomic mouth formation, and amphioxus and olfactoreans acquired new methods of mouth formation after dorso-ventral inversion. Amphioxus opens mouth by using coelom and its canal, which has a common origin with ambulacrarian coelomic pore-canals (asterisks). Olfactoreans developed a placode or its equivalent at the anterior extremity to form a stomodaeum

The present study confirms the close association of the mouth development with the Hn in amphioxus. This may support a serial homology between the amphioxus mouth and gill slits [11] as each paired gill slit also associates with branchial nephridium (Bn) [46]. Like the Hn/OMV complex, Bns open into the pharynx, and gills form through this opening by perforating ectoderm-mesoderm and endoderm-mesoderm bi-layered membranes (Fig. 8) [46]. Despite this similarity, we favor the idea that the amphioxus mouth and ambulacrarian coelomic pore-canal originate from a shared genetic background established in a common ancestor (Fig. 9) because the developmental timing relative to surrounding tissues shows a large disparity between the Hn and Bns. The Hn develops during early larval stages, whereas all Bns develop during metamorphosis and onward as paired gill openings. The similarity between the amphioxus mouth and paired gill openings rather suggests that these developmental mechanisms retain an ancestral state that offers an insight into the origin of pharyngeal gill slits in the deuterostome ancestor, a question that remains unexplored.

Dorso-ventral inversion is not only an issue of body axes, but also of mouth formation. Gene expression analyses suggest that a dorso-ventral inversion occurred in deuterostomes, possibly in a chordate ancestor [8]. When chordates acquired dorsal structures such as the notochord and epithelial neural tube, they utilized a signaling center at the dorsal margin of the blastopore with GRN including *nodal*, *goosecoid*, *chordin*, and *brachyury*. Interestingly, the chordate dorsalizing GRN is comparable to that determining the oral side of sea urchin embryos [47]. If this similarity is related to the dorso-ventral inversion, chordates needed a new means of mouth formation. Gastrulation in chordates permits new mouth formation by having the developing primitive gut underlie the epiblast (outer layer), especially in the anterior region, contrary to ambulacrarian gastrulation, in which the primitive gut does not fill the blastocoel or rapidly differentiate a protocoel at the anterior end in some enteropneust embryos [48, 49]. To open a new mouth, olfactoreans (urochordates + vertebrates) developed an anterior placode or stomodaeum under a median Pitx function [20, 38], whereas amphioxus utilizes an ancestral coelomic canal coupled with the early Pitx function on the anterior left side, probably owing to the inability to form placodes [50]. In mouth formation, the Nodal-Pitx signaling unit has likely been co-opted variously as this unit is also utilized in the molluscan stomodaeum [51]. The amphioxus mouth does not represent an ancestral condition of the vertebrate mouth, but instead may display a close relationship to the deep origin of gill openings in deuterostomes.

## Conclusions

We have studied the development of the amphioxus mouth that initially opens on the left lateral side of the pharynx. The unusual location of the amphioxus mouth is caused by unique development that involves the coelomic mesoderm. We conclude that the opening of the amphioxus mouth is mediated by a coelomic vesicle (OMV) that develops from the posterior ventral corner of the left first somite in association with the Hatschek nephridium. This OMV development is controlled by the Nodal-Pitx unit that gives rise to the left-right asymmetrical development. The developmental pattern of the amphioxus mouth leads us to hypothesize on the common evolutionary origin of the amphioxus mouth and ambulacrarian pore-canals. This unique mouth is parsimoniously regarded an apomorphic character most likely acquired at the appearance of the amphioxus lineage and has no relation to the olfactorean mouths. As the olfactorean group also develops a mouth under a GRN different from that in ambulacrarians, which is likely ancestral in chordates, we also hypothesize that new chordate mouths were acquired in relation to the dorso-ventral inversion that occurred in the last common ancestor of chordates.
